# Revealing General Patterns of Microbiomes That Transcend Systems: Potential and Challenges of Deep Transfer Learning

**DOI:** 10.1128/msystems.01058-21

**Published:** 2022-01-18

**Authors:** Maude M. David, Christine Tataru, Quintin Pope, Lydia J. Baker, Mary K. English, Hannah E. Epstein, Austin Hammer, Michael Kent, Michael J. Sieler, Ryan S. Mueller, Thomas J. Sharpton, Fiona Tomas, Rebecca Vega Thurber, Xiaoli Z. Fern

**Affiliations:** a Department of Microbiology, Oregon State University, Corvallis, Oregon, USA; b Department of Pharmaceutical Sciences, Oregon State University, Corvallis, Oregon, USA; c School of Electrical Engineering and Computer Science, Oregon State University, Corvallis, Oregon, USA; d Department of Statistics, Oregon State University, Corvallis, Oregon, USA; e Instituto Mediterráneo de Estudios Avanzados, IMEDEA, Esporles, Balearic Islands, Spain; University of Minnesota

**Keywords:** deep learning, embeddings, machine learning, microbial ecology

## Abstract

A growing body of research has established that the microbiome can mediate the dynamics and functional capacities of diverse biological systems. Yet, we understand little about what governs the response of these microbial communities to host or environmental changes. Most efforts to model microbiomes focus on defining the relationships between the microbiome, host, and environmental features within a specified study system and therefore fail to capture those that may be evident across multiple systems. In parallel with these developments in microbiome research, computer scientists have developed a variety of machine learning tools that can identify subtle, but informative, patterns from complex data. Here, we recommend using deep transfer learning to resolve microbiome patterns that transcend study systems. By leveraging diverse public data sets in an unsupervised way, such models can learn contextual relationships between features and build on those patterns to perform subsequent tasks (e.g., classification) within specific biological contexts.

## PERSPECTIVE

It is now apparent that microbiomes frequently mediate the dynamics and functional capacities of environmental and biological systems and in so doing can impact system health and homeostasis (1). Diverse sources of biotic and abiotic variation can affect these microbial communities, which in turn can reciprocally impact the health and functioning of their host or habitat. In this era of rapid, human-induced environmental change, our ability to manage biological and environmental systems may very well depend upon our understanding of how microbes interact with one another as well as with their host or environment and how exogenous variation (i.e., variation external to the host or environment microbiome itself) impacts these interactions. To propel this understanding, microbiologists have developed technologies that catalog and quantify measurable features of the microbiota, their host, and the environment. From environmental DNA sequencing to metabolomics, microbial ecologists increasingly generate, analyze, and integrate massive multi-omic data sets to characterize communities, understand the mechanisms they use to interact and drive their environment, and determine how these community components respond to environmental changes (2). In doing so, microbial ecologists often focus solely on their specific data sets and do not leverage the vast repository of publicly available microbial data to extract complex but generalizable patterns in relation to specific scientific questions.

Researchers currently apply powerful statistical and machine learning methods to a specific microbiome data set to identify microbial features (i.e., the measurable variables of a system: taxa, genes, metabolites) that stratify groups of samples or that explain the variation of a continuous covariate across samples (2–7). Traditional methods may also use summary metrics like beta-dispersion and alpha- and beta-diversity to characterize microbial community patterns (8). However, neither the single feature approach nor the community summary statistic approach incorporates patterns across studies or systems, nor do they take into account the interdependencies between features. Thus, they will fail to capture basic principles governing how microbial communities assemble, diversify, and respond to environmental variation.

Currently, meta-analyses are used to apply these traditional analysis approaches across studies. However, while meta-analyses of microbiome data sets generally support relationships between microbiome changes in community structure or function and disease observed in individual studies (9, 10), these interactions are often relatively weak and confounded by interstudy and individual microbiome variation (11–14). This may be due to a limitation in the traditional analysis methods, which treat each study independently and consequently may miss key insights into a system’s more generalizable biological properties. To overcome these limitations, prior work has sought to robustly link specific microbiome features to outcomes and phenotypes across studies (11, 15). These studies go beyond traditional meta-analyses by identifying generalizable properties of microbial communities using network analysis or by accounting for noise across multiple data sets when developing predictive models. Such work shows that (i) there are generalizable patterns among diverse microbiomes that could be better exploited and (ii) many of these patterns or structures may be undetectable within individual studies due to the contextual nature of the patterns and limitation of the study sample size.

We believe deep transfer learning can expand on these approaches and is particularly well suited to generalize patterns of microbial ecology across studies and biological systems. We and others are leveraging deep learning methods (Table 1) both to capture microbe-microbe and microbe-environment interactions across systems and to generate models and transformed features informed by aggregations of all available data. Deep learning approaches are specifically designed to analyze diverse, high-dimensional data in ways that can detect complex patterns of association between multiple features and covariates of interest. As a result, deep learning approaches can discover associations between a feature and a covariate that are contextually dependent upon other features in the system. Moreover, deep neural networks have proved capable of transfer learning, the process of learning generalizable patterns from diverse data and then fine-tuning that general model on study- or system-specific tasks (16–18). By developing deep learning approaches that resolve shared patterns across studies, researchers can draw novel insights about well-studied communities and aid discovery in understudied environments.

**TABLE 1:** Lexicon of key terms and their definitions

Term	Definition
Machine learning	Data analytics methods that use a variety of algorithms learning from available data to optimize the parameters of models, which are often predictive of classes if used in a supervised context.

Deep learning	A class of machine learning algorithms originally inspired by the brain. Deep models have multiple layers that sequentially process inputs, have high parameter counts, and use large amounts of data to train. In training, deep models learn to extract high-level features that are useful for both the trained model and potentially other models down the analytical pipeline.

Pretrained models	Models trained on a “pretraining” objective for which there is a very large amount of data. Such models can then be adapted to a “downstream” objective for which there are far fewer data and are often able to leverage information learned from the pretraining data to more easily perform the downstream objective.

Transformer models	A specific type of deep learning model originally proposed for language but later applied to many other domains, including vision, graphs, and sets.

Embeddings	Higher-level features derived from deep learning algorithms. They can efficiently represent statistical patterns in microbial communities, aiding in data processing for such models.

Transfer learning	The process of training a model on one task/data set and then fine-tuning it to perform a different task on a different data set is known as transfer learning.

Unlabeled data	Use of microbiome-related data (may be of different data types) without requirement for specific metadata associated with a given prediction task.

Language model	Models based on algorithms originally developed by the field of natural language processing, trained to model natural language and estimate the probability of a given word appearing in a certain context.

Task-specific model	Models developed to predict a specific set of features or outcomes and almost always trained on a prediction task with labeled data.

In particular, we advocate using deep learning models that have already proven adept at transfer learning in natural language processing (NLP) to better understand baseline interactions present in microbiome data (19–21). There exist easily drawn parallels between natural language data and microbiome data, namely, that documents are equivalent to biological samples, words to taxa, and topics to microbial neighborhoods (22–24). While other language-inspired algorithms such as topic modeling methods have been employed to identify latent variables in microbiomes, deep learning approaches offer the unique advantage that they scale with the amount of data available, allowing our understanding and our predictive models to scale alongside the genomic revolution (25).

One simple NLP algorithm is embedding, where a low-dimensional space is learned that preserves information about the cooccurrences between features. Embedding-based analysis of gut microbiomes has previously enabled a more accurate and generalizable differentiation between microbiomes associated with inflammatory bowel disease and healthy gut microbiomes than does analysis based on individual taxon counts (26). It was also observed that a word embedding algorithm applied to 16S k-mers resulted in meaningful numeric feature representations that both bolstered downstream classification performance and offered insight into the correspondence of microbial taxa to particular body sites (27).

**FIG 1:**
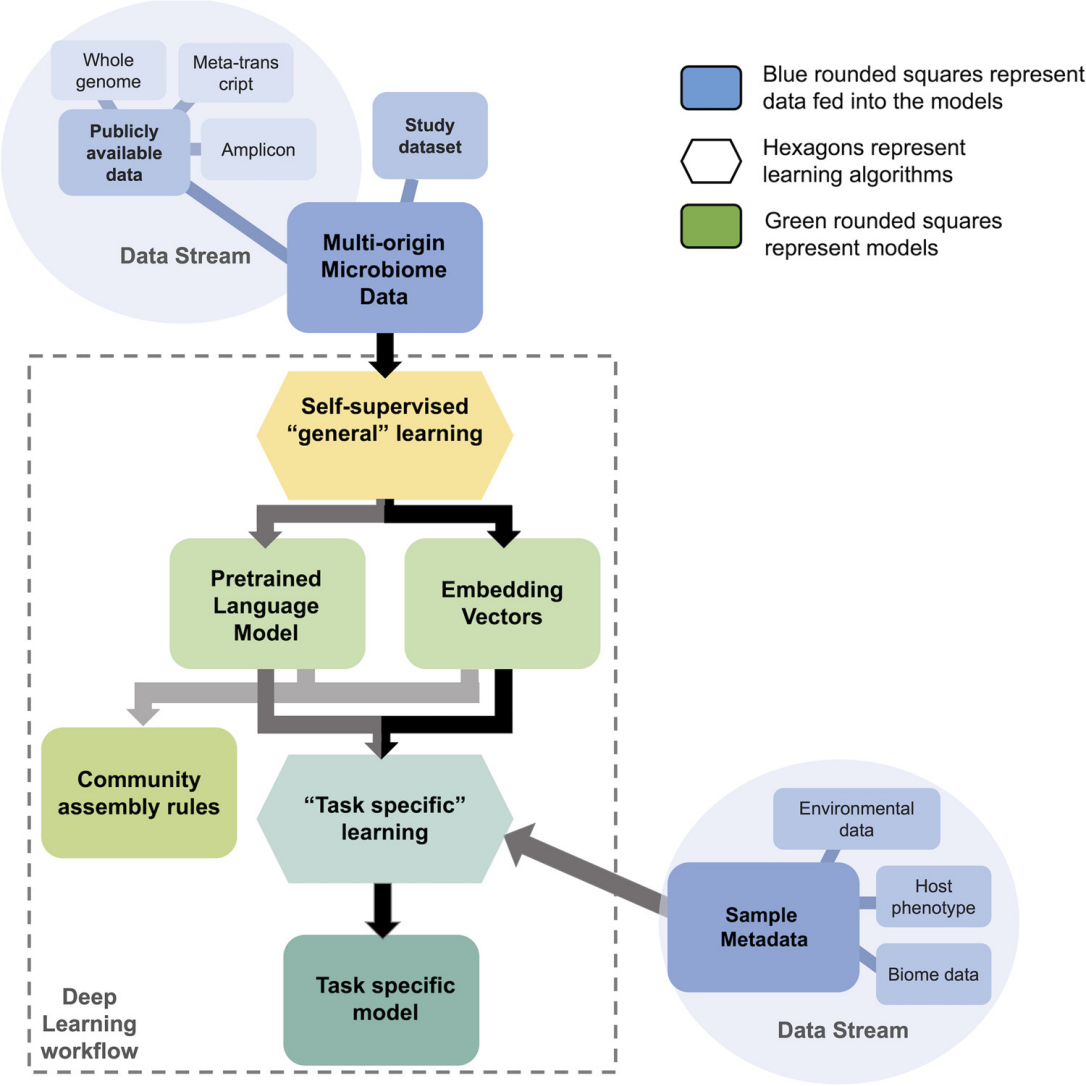
Workflow of microbial data transformation using deep learning approaches used to capture cross-system properties and apply to a specific task. Hexagons represent algorithms, fed data (in blue) and producing models (squares). Measured microbial features from publicly available data sets are input into the deep learning algorithms (in yellow) to produce pretrained models constituting new features (in green). During pretraining, a model is trained to reconstruct original input from distorted or partial input, using unlabeled data. The process outputs a trained model and/or a set of feature embeddings (which may be interpreted to represent community assembly rules and microbial ecology principles). The general model may then be fine-tuned on metadata (study data set in blue) to answer a biome- or system-specific question of interest. The process of training a model on one task/data set and then fine-tuning it on another task/data set is known as transfer learning.

Another powerful approach is to pretrain a model using “self-supervised” learning strategies on a large, diverse corpus (21, 28). In natural language, this can be achieved by (i) blocking out words from the input text and asking the model to recreate the hidden portion or (ii) asking the model to generate the next word in the sentence. This forces the model to identify properties in the input data that are useful for inferring the missing information and can lead the model to learn linguistic and semantic patterns that govern the composition of natural text beyond simple word cooccurrences (e.g., grammatical rules, semantic relationships, and other statistical patterns that aid in understanding text). Analogously, self-supervised learning can be applied to learn a “language” model of the microbiome, which captures the interactions among different microbial species or metabolic processes and can be used to understand shared composition rules of microbial communities. The pretrained language models can encode each word, or measured bacterial feature, into an embedding, which can subsequently be used for supervised prediction tasks (to differentiate two systems, or the same perturbation across two systems, for example) (Fig. 1) with greatly reduced dimension, hence reducing the risk of overfitting. These models offer distinct advantages over conventional methods because they use large data sets of bacterial features from diverse species and environments. Such models may be used to generalize across microbial systems to reduce input dimensionality and find useful latent properties in microbial communities.

There are important distinctions between natural language and microbiome data. Language has a natural sequential structure that is not present in most omics-based microbiome data. Comparatively, microbiome data also has rich biological information about taxa and functions that helps generate ecological and evolution inferences into whole-system dynamics. Such distinctions offer exciting opportunities for new innovations to adapt NLP models to microbiome data, similar to what has been done in computer vision (29) and learning with graphs (30) and sets (31) (Table 2).

**TABLE 2:** Nonexhaustive list of relevant examples of deep learning algorithms and their ecological representations, when applicable^*a*^

Deep learning algorithm	Description	Application to microbial ecology	Papers
Autoencoder	Condense a long vector of input features into a dense mathematical space and then validate by regenerating the input from the condensed space.	16S amplicon, gene, metabolite, or protein counts can be condensed, thereby defining latent variables that drive observed feature counts. Ecological representation: latent variables may represent environmental factors like nutrient availability or pH that dictate observed microbial features.	“Using autoencoders for predicting latent microbiome community shifts responding to dietary changes” (37); “DeepGeni: deep generalized interpretable autoencoder elucidates gut microbiota for better cancer immunotherapy” (38); “Utilizing longitudinal microbiome taxonomic profiles to predict food allergy via Long Short-Term Memory networks” (39); “Predicting microbiomes through a deep latent space” (40); “DeepMicro: deep representation learning for disease prediction based on microbiome data” (41)

Convolutional neural networks	A class of models that use convolution kernels or filters that slide along input features to learn features between input segments with a given spatial relationship	Integrate spatial information about relative and global microbial locations within a system. Interpret nucleotide text. Ecological representation: metabolic relationships dependent on location within a system. Motif function dependent on location relative to other motifs.	“TaxoNN: ensemble of neural networks on stratified microbiome data for disease prediction” (42); “Learning, visualizing and exploring 16S rRNA structure using an attention-based deep neural network” (43)

Long short-term memory (LSTM)	A class of deep neural networks that process data sequentially. They have a memory that updates every time the network processes a sequence entry.	Learn patterns from microbial genetic sequence (e.g., shotgun metagenomics or transcriptomics) which hold information about gene function. Represent longitudinal count data and changing microbiome dynamics along a nutrient or time gradient. Ecological representation: gene clusters of interreliant genes. Changes in specific taxon abundances over time are indicative of system state.	**“Transfer learning improves antibiotic resistance class prediction” (**33**)**; “Utilizing longitudinal microbiome taxonomic profiles to predict food allergy via Long Short-Term Memory networks” (39); **“Shedding light on microbial dark matter with a universal language of life” (**34**)**

Embedding	Learn word vectors so the inner product of vectors *i* and *j* reflects the cooccurrence probability for words *i* and *j*. Specific example: GloVe.	Consider taxon or any microbial feature cooccurrences. Ecological representation: metabolic corporations or partnership.	“GloVe: global vectors for word representation” (44); **“Decoding the language of microbiomes using word-embedding techniques, and applications in inflammatory bowel disease” (**26**)**

Continuous bag of words	Generate one static embedding per word. Training algorithm captures semantic and lexical information of each word based on that word’s context—i.e., its neighboring set of words. Specific example: Word2vec.	k-mer representations of sequences could be embedded in such a way that their context is preserved. Ecological representation: conserved motifs (k-mers) represent evolutionary and possibly functional similarities across sequences.	Word2vec (45); “Learning representations of microbe-metabolite interactions” (46)

Transformer	Guess the next word or part of a sentence based on all the words present in a sentence. Mask input text tokens, then train the model to predict original tokens from unmasked context. Distort text by replacing input text tokens with fake but plausible substitutions. Then train model to identify the fake tokens based on the surrounding context. Specific examples: BERT, ELECTRA	Provide a view of a sample by considering all neighbors. Identify key microbial features association within samples. Ecological representation: community assembly and/or metabolic cooperation at a higher degree.	“BERT: pretraining of deep bidirectional transformers for language understanding” (21); “ELECTRA: pretraining text encoders as discriminators rather than generators” (47)

a Papers highlighted in bold engage in transfer learning where an independent pretraining task and data set are used to build a model, which is then fine-tuned on a different prediction task and data set.

While the application of deep learning to study microbiomes holds great promise, there exist significant challenges. Deep learning requires large amounts of data. For example, BERT, a commonly used pretrained language model for natural text, was trained on 3.3 billion words (23). While the scientific community has been generating microbiome data exponentially, these data are often not publicly available or remain unprocessed or uncurated. For example, while NCBI counts around 690 million sequence records (as of August 2021), the vast majority of records do not contain the metadata information sufficient to include in training data sets. Conversely, well-curated databases often lack sufficient depth of records. For example, databases like Qiita, which aim to enable cross-system meta-analyses of omics data, contain on the order of tens of millions of individual sequence records (e.g., amplicon sequence variants [ASVs] from 16S amplicon studies) from a few hundred thousand (160,000 to 200,000) samples of various omics types (32). Because of this, transfer learning in the microbial space thus far has largely focused on using pretraining tasks that do not require curation, such as predicting the next segment of genetic sequence or the next amino acid of a protein sequence, from minimally processed reads (33, 34). Examples of transfer learning using predefined microbial features (e.g., ASVs, genes, or metabolites) are rare. However, our first attempt determined that approximately equal numbers of features (ASVs) and samples were sufficient to capture meaningful information in embedding vectors while drastically reducing the dimensionality from ∼23,000 ASVs to 100 dimensions (26).

Given the complexity and noisiness of biological systems, integration of different omics data representing different processes will be key in the development of more generalizable models that transcend individual systems. Accordingly, understanding the biological relationships between these data types and what limitations and insights they provide into biological processes is a large and important challenge. The scientific community needs to (i) ensure metadata is available alongside omics; (ii) adopt a common ontology for metadata type and sequencing data type, such as those being developed by the National Microbiome Data Collaboration (35); and (iii) allow universal and easy access to those data. There is an urgent need to organize data catalogs, or at least to categorize any new data generated during published research. These efforts should follow principles such as the FAIR data standards (36) of Findability, Accessibility, Interoperability, and Reuse of digital assets. In addition to requiring large amounts of data, deep models are often difficult for humans to understand. Although the learned patterns of neural network models can detect useful and general statistical patterns in microbial data, interpreting those models remains challenging. How can we extract information from properties or dimensions that humans have not conceptually defined yet? Work on interpreting neural network models is well under way in the field of computer science, and cross-disciplinary partnerships that leverage that work are becoming increasingly valuable, especially as the amount of microbiome omics data grows year over year.

To conclude, we call upon the scientific community to collaboratively accelerate these endeavors: the algorithms developed in various branches of computer science, including natural language processing and explainable artificial intelligence (AI), offer us unprecedented opportunities to learn from complex and massive data sets and gain transformative insight into the rules that govern how microbial communities assemble, diversify, and respond to environmental variation. This effort is particularly relevant in an age where our ability to synthesize data on model systems outstrips our ability to sample across every microbial system on Earth.
